# Intraoperative and Postoperative Intraocular Lens Opacifications: Analysis of 42545 Cases

**DOI:** 10.1155/2021/1285947

**Published:** 2021-12-06

**Authors:** Xiaochen Wang, Xiaoming Wu, Yunhai Dai, Yusen Huang

**Affiliations:** ^1^Qingdao Eye Hospital of Shandong First Medical University, Qingdao, China; ^2^State Key Laboratory Cultivation Base, Shandong Provincial Key Laboratory of Ophthalmology, Eye Institute of Shandong First Medical University, Qingdao, China

## Abstract

**Purpose:**

To assess the types and causes of intraocular lens (IOL) turbidity in a tertiary eye center. *Setting*. Qingdao Eye Hospital of Shandong First Medical University, Qingdao, China.

**Design:**

Retrospective case series.

**Methods:**

Patients who underwent uncomplicated phacoemulsification and IOL implantation for cataract between January 2015 and December 2019 were included. Medical records were reviewed of participants with intraoperative or postoperative IOL opacification for clinical data, artificial crystal materials, and causes of the opacification.

**Results:**

A total of 42545 IOLs were implanted in the five years, comprising 25471 (66.0%) hydrophilic IOLs, 11881 (27.9%) hydrophobic IOLs, and 2601 (6.1%) hydrophilic-hydrophobic acrylic IOLs. Among the operated eyes, 14 eyes (13 patients) experienced IOL opacification, which was permanent for 10 IOLs, including 7 (0.6%) hydrophilic IOLs (860UV) and 3 (0.2%) hydrophilic-hydrophobic acrylic IOLs (L-312). The mean interval between surgery and diagnosis of permanent opacification was 34.4 ± 18.4 (SD) months (range, 12 to 59 months). Permanent IOL clouding led to a statistically significant reduction in best corrected visual acuity (mean, 0.64 ± 0.4 logMAR; *P* < 0.004). Acute IOL clouding occurred in four eyes during the implantation of a hydrophilic-hydrophobic acrylic IOL of L-312, 809M, or 839M and returned to transparency several hours later. All four procedures were performed in winter, with the mean outside temperature being −5.75°C.

**Conclusions:**

The rate of IOL opacification was 0.03%. Both delayed postoperative and acute intraoperative opacifications occurred with various characteristics in IOLs made of different materials and designs. Clinicians should be aware of this risk for cataract surgery.

## 1. Introduction

Since the invention of intraocular lenses (IOLs) in 1949, biomaterials used in their production have continually improved. The new soft IOLs are manufactured in a variety of designs and materials including silicone, hydrophobic acrylic, and hydrophilic acrylic [1]. These IOLs possess their specific advantages. Hydrophilic acrylic IOLs have higher tissue compatibility due to a high water content and higher uveal biocompatibility compared with hydrophobic acrylic IOLs [2, 3], while hydrophobic acrylic leads to a lower rate of anterior and posterior capsule opacification than hydrophilic acrylic [4].

In recent years, however, turbidity has been occasionally found in both hydrophilic and hydrophobic IOLs during or after cataract surgery, even resulting in blurred vision and IOL explantation [5–7]. Such opacifications may be related to the patient's condition, manufacturing process, IOL storage, surgical technique and adjuvants, or their combination [8–10]. Calcification could arouse irreversible IOL opacification, especially in hydrophilic IOLs [11], opacities of which differ from those typical of hydrophilic lenses and appear as single or multiple vacuole-like glistening microcavities. Moreover, considering the reversibility of opacification and the short interval between IOL implantation and occurrence of clouding in some cases, temporary lens epithelial cell outgrowth, delayed atypical toxic anterior segment syndrome [12, 13], and even temperature volatility can be the reasons.

This complication of IOL opacity has been noticed since 2010 at our institution and subjected to statistical analysis and laboratory research [14, 15]. Herein, we documented a cluster of cases of irreversible opacification associated with hydrophilic IOLs and hydrophilic-hydrophobic acrylic IOLs, as well as four cases of transient clouding, during IOL implantation.

## 2. Patients and Methods

This retrospective case series study was performed at the Qingdao Eye Hospital of Shandong First Medical University, a tertiary eye center in Qingdao, China. We counted the number of IOLs used in our institution from January 2015 to December 2019 and analyzed the proportion of hydrophobic, hydrophilic, and hydrophilic-hydrophobic copolymer IOLs. Data of patients receiving implantation of these IOLs during cataract surgery were collected, including the patient's condition, date of IOL implantation, IOL serial number, IOL type, intraoperative and postoperative complications, and additional intraocular surgical procedures.

The diagnosis of IOL opacification was based on a careful slit-lamp examination. Consecutive patients with intraoperative or postoperative IOL clouding were included in the statistical analysis, and some of them were referred for IOL explantation to our institution. The interval between IOL implantation and diagnosis of opacification was evaluated. Patients who presented with clinically significant impairment in visual acuity were proposed to have the IOL explanted.

All statistical analyses were performed with SPSS® statistical software, version 20 (IBM SPSS Statistics for Windows, IBM Corporation, Armonk, NY). Quantitative variables were compared using the nonparametric Kruskal–Wallis test. Significance was assumed when the *P* value was less than 0.05. All BCVA were converted into a logMAR unit for the statistical analysis.

## 3. Results

A total of 42545 IOLs were implanted during cataract surgery through the 5 years, comprising 25471 (66.0%) hydrophilic IOLs, 11881 (27.9%) hydrophobic IOLs, and 2601 (6.1%) hydrophilic-hydrophobic acrylic IOLs. Among all operated eyes, 14 eyes (0.03%, 13 patients) suffered IOL opacification. The mean age of the 13 patients at the time of cataract surgery was 71.5 ± 9.7 (SD) years (range, 47 to 79 years). The involved IOLs were 860UV (US), L-312 (Oculentis), 809M (Carl Zeiss), and 839M (Carl Zeiss). The clouding occurred postoperatively and remained permanent in 10 eyes and was transient intraoperatively in 4 eyes.

**Figure 1:**
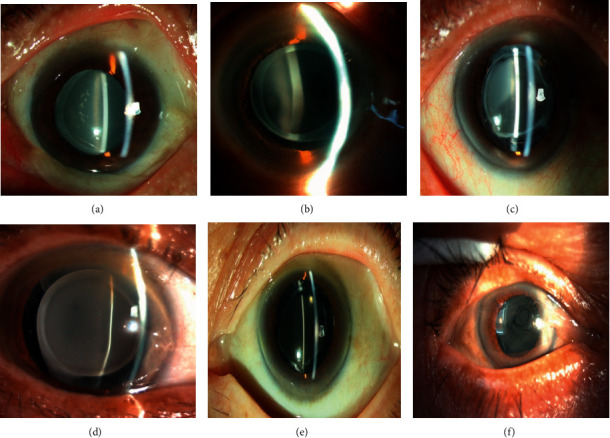
Photographs of the distinct case of IOL opacification. (a)–(e) The postoperative opacification was 860UV IOLs, (f) the opacification IOL was L-312.

Clinical data of patients with permanent clouding are summarized in Table 1. The delayed opacification developed in 7 (0.6%) of 11619 implanted 860UV IOLs and 3 (0.2%) of 1882 implanted L-312 IOLs. By slit-lamp microscopy, a granular and diffuse appearance was observed on the front and back surfaces of the 10 lenses (Figure 1). Patients 1 and 8 also suffered posterior capsule opacity. In these 9 patients, none had intraocular surgery other than cataract surgery before the occurrence of IOL opacification. Nonproliferative diabetic retinopathy was found in 1 patient (11.1%). Major systemic conditions included arterial hypertension (2 patients, 22.2%), diabetes mellitus (2 patients, 22.2%), and renal insufficiency (1 patient, 11.1%). The mean best corrected visual acuity (BCVA) after cataract surgery was 0.18 ± 0.1 logMAR. All patients had a smooth surgical procedure with the IOL placed in the capsular bag and an uneventful postoperative course.

The mean interval between implantation of the IOLs and the diagnosis of opacification was 34.4 months ±18.4 (SD) (range, 12–59 months). All 9 patients had the opacified IOLs explanted during January 2019 and December 2019. The mean BCVA before IOL exchange was 0.82 ± 0.49 logMAR, significantly lower than that after phacoemulsification (mean, 0.64 ± 0.4 logMAR) (*P* < 0.004). During explantation, the opaque lens was viscodissected and removed through a 6.0 mm scleral incision at the 12 o'clock position. An angulated 3-piece hydrophobic acrylic IOL was implanted with a fixation in the ciliary sulcus in 3 eyes, a 1-piece hydrophobic acrylic IOL was implanted in the capsular bag in 1 eye, and a 1-piece hydrophilic acrylic IOL was implanted in the capsular bag in 4 eyes. Anterior vitrectomy was performed as necessary.

**Figure 2:**
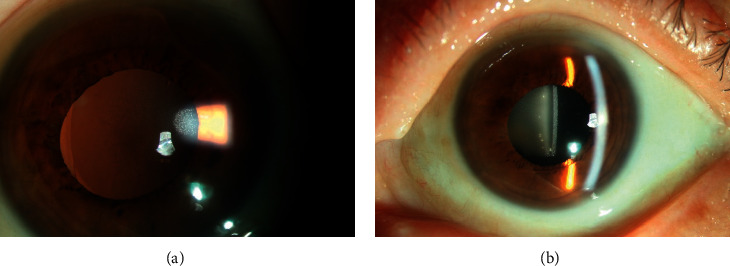
Clinical slit-lamp photograph of bilateral opacification of L-312 IOLs. Note the granular and diffuse opacification of the IOLs.

Of the 9 patients, 7 (77.8%) had bilateral cataract surgery, including 5 (71.4%) implanted with 860UV IOLs and 2 (28.6%) with Lentis L-312 IOLs in both eyes. Six patients had no detectable opacification in the contralateral eye during the follow-up. Bilateral opacification occurred in 1 patient with implantation of Lentis L-312 IOLs (Figure 2), who was 76 years old and had a history of diabetes, arterial hypertension, and renal insufficiency.

**Figure 3:**
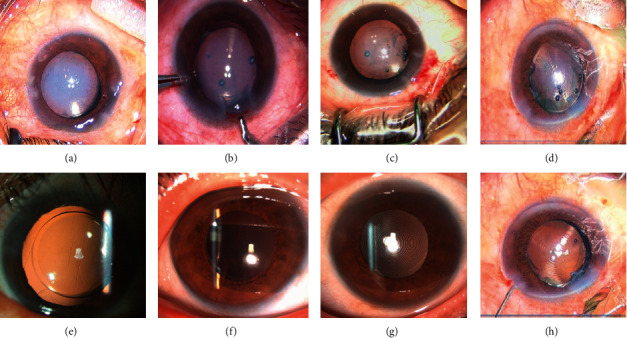
Intraoperative IOL opacity. (a)–(d) The clouding of the optical region immediately after IOL implantation. (e)–(g) The transparent IOL at 1 day after surgery. (h) No opacification after irrigation.

Clinical data of 4 patients (4 eyes) with acute IOL opacification are shown in Table 2. The IOLs transiently became opaque in varying degrees during surgery (Figures 3(a)–3(d)). When such an opacity was first encountered, the surgeon replaced the L-312 IOL immediately. Afterwards, both the bifocal (809M) and trifocal (839M) IOLs presented the same problem. The lenses were not removed but constantly monitored, and they recovered transparency after several hours, resulting in no permanent structural or mechanical change (Figures 3(a)–3(d)). The four surgical procedures were performed in winter, when the outdoor temperatures were below zero (mean, −5.75°C). In patient 1, the IOL had been stored at room temperature for 2 hours following 1-hour transportation at an outside temperature of −3°C. The opacification was found to be alleviated after 2 hours. In patient 4, the lens was delivered to the theater at an outdoor temperature of −4°C and 3 hours ahead of the implantation. After irrigation, the opacified IOL started to clear. We noted that the lower the outdoor temperature and the shorter the indoor placement time, the more seriously the IOLs were affected.

## 4. Discussion

IOL opacification which could happen intraoperatively or postoperatively is a rare but serious complication of cataract surgery. Our study presented a comparatively large series of this phenomenon, concerning several IOL designs and materials available. Late postoperative opacification of IOLs might lead to a severe reduction of visual function and interfere with the fundus examination. Silicone and acrylic (hydrophilic and hydrophobic) are the principal materials used for manufacturing foldable IOLs, which can be inserted into the eye through a small self-sealing incision. Opacification in all these materials at different degrees has been evaluated in the past decades, although it is more common with hydrophilic acrylic IOLs than with hydrophobic acrylic or silicone IOLs, owing to the calcification issue [4–10].

Silicone IOLs as the first generation of foldable lenses have been known to undergo brownish discoloration and central haze within 6 weeks after surgery, which may be attributed to light scatter from a layer of water vapor within the IOL that has diffused into the silicone material [11, 12]. Hydrophobic acrylic lenses (Acrysof, Alcon, US) have been demonstrated to show glistenings, which are actually refractile microvacuoles in the IOL optic forming at an aqueous environment, resulting rarely in blurred vision. In vitro studies [13, 16] have suggested that IOL manufacturing techniques, temperature change of the surrounding environment, and IOL packaging might be the causes for this vacuolation. Postoperative opacification of modern hydrophilic acrylic IOLs has also been reported due to primary calcification, with the Hydroview model H60m (Bausch & Lomb, Rochester, NY, USA), SC60B-OUV (Medical Developmental Research, Clearwater, FL, US), ACRL-60 (Ophthalmed Inc.), MemoryLens (Ciba Vision, Duluth, GA, US), and AquaSense (Ophthalmic Innovations International, Ontario, CA, USA) involved [16–19]. Our group previously disclosed opacification in 3 Hydroview model H60M IOLs, which originated from calcium and phosphorous, as confirmed by energy dispersive X-ray and scanning electron microscopy [14]. We also described the density/severity of glistenings in the hydrophobic acrylic AcrySof IOLs MA60MA (Alcon) [15]. In the current series, hydrophilic acrylic 860UV IOLs were found to have a similar pattern of opacification as the prior report. The Lentis L-312 IOL, which is made of a “Hydrosmart” hydrophilic biomaterial with a high water content and a hydrophobic surface, was also shown to have irreversible clouding, confirming that hydrophilic IOLs with a hydrophobic surface are not immune to opacification [20]. Bompastor-Ramos et al. [20] identified calcium hydroxyapatite as the predominant mineral, which was responsible for the opacification of the LS-502-1 IOL. Gurabardhi et al. [21] described a few designs of calcified IOLs manufactured by Oculentis, concluding that a manufacture issue might be the reason for IOL opacification. We also found most of the 860 UV IOLs with turbidity were made in 2017, and the related L-312 IOLs were all made in 2015. The complication can be suspected to have something to do with the materials and production process.

Furthermore, there were four patients experiencing acute IOL clouding in our study. The IOLs were all hydrophilic acrylate lenses with a hydrophobic surface. The copolymer materials seem to be susceptible to temperature change and, thus, liable to suffer temporary clouding. However, IOLs made of a single material, such as a completely hydrophobic or hydrophilic material (Softec HD, AO, and iSert251) were not found to have any acute clouding because of the sudden temperature fluctuation.

Regarding the patient's conditions, it is not clear whether alteration of the blood-aqueous barrier is a factor in inducing inflammation that results in opacification of the hydrophilic material. A high percentage of diabetes [22] and uveitis was reported in patients with late postoperative opacification of hydrogel lenses, although no clear risk factor could be identified. Kim et al. [7] described two cases of completely reversible opacification of hydrophobic acrylic IOLs (Tecnis ZCB00) due to either temporary lens epithelial cell outgrowth or delayed atypical toxic anterior segment syndrome. In this series, two patients had hypertension and coronary heart disease, two patients had stable diabetes, and one patient had renal insufficiency. It is suggested that systemic comorbidity, particularly diabetes and metabolic disease, and other intraocular surgery and/or therapeutic treatment following IOL implantation may contribute to IOL opacification, although no strong correlation has been found.

Compared with the materials and properties of the IOLs, the importance of their storage and transport is often overlooked. All four cases of acute clouding of hydrophilic IOLs occurred in winter. There was no heat preservation during the transportation, and the IOLs were placed in the operating room for less than 24 hours. We hypothesize that, after storage in low temperature (below zero) and immediate injection into the eye at approximately 37°C, microbubbles and fogging may be formed when the cool IOL contacts with the aqueous humor. Once the microbubbles reach equilibrium in the aqueous humor and the IOL, the clouding would disappear spontaneously. Also noteworthy, these IOLs have an instruction for presurgical storage in the instructions for use; just like the L-312 lens, it is suggested to be transported at an environment temperature between 5°C and 50°C and stored at room temperature for at least 1 hour before use. Also, in the AT LISA 809M crystal instruction, room temperature is indicated at the time of surgery to avoid temporary clouding of the lens optic after implantation.

In conclusion, we summarized a series of opacification in different types of IOLs attributable to various reasons. Our results highlight that neither hydrophilic acrylic IOLs nor hydrophilic IOLs with a hydrophobic surface are immune to this complication. Clinicians should be cautious when selecting IOLs. It is of utmost importance that materials used in IOL manufacturing and packaging ensure long-term transparency after the IOL is implanted. Since IOL changes are obviously of multifactorial causation, further studies are required. Attention should also be given to the intraoperative acute IOL opacity. Continued efforts are needed to disclose the underlying mechanisms of opacification in IOLs.

## Figures and Tables

**Table 1:** Characteristics of the patients with postoperative IOL opacification.

Case	IOL type	IOL serial number	Sex/age (Y)	Date of implantation	Time interval (Mo)^#^	BCVA		Associated general conditions
						Before	After	
1	860UV	—	M/68	26/2/2017	18	20/25	20/200	—
2	860UV	O-28416001-029	M/75	09/05/2017	20	20/40	20/63	—
3	860UV	O-10717012-022	M/76	26/12/2017	23	20/25	20/50	—
4	860UV	—	M/73	09/02/2017	48	20/25	20/32	—
5	L-312	91310521013	M/47	01/12/2015	56	20/25	20/200	Diabetes
6	860UV	O-04717025-033	F/73	13/11/2017	24	20/32	20/125	Hypertension, cardiopathy
7	860UV	O-21116050-006	F/76	16/01/2017	12	20/32	20/200	—
8	860UV	O-04817001-005	F/79	13/07/2017	27	20/25	20/50	Hypertension
9	L-312	91307941002	F/71	08/10/2015	59	20/32	20/50	Diabetes
	L-312	91307941004	F/71	26/09/2015	58	20/32	20/100	Renal insufficiency

IOL = intraocular lens; ^#^time interval between implantation and diagnosis of opacification. Before, before IOL opacification; after, after IOL opacification.

**Table 2:** Characteristics of the patients with transient IOL opacification.

Case	IOL type	IOL serial number	Sex/age (Y)	Opacification date	Temperature		BCVA		Time interval (ho)^*∗*^
					Outdoor (°C)	In room (°C)	Before	After	
1	L-312	91306104022	F/64	17/12/2015	−3	21	20/40	20/25	2
2	809M	1S1710870057	M/54	09/01/2018	−8	21	20/50	20/20	1.5
3	809M	1S1713970329	F/62	30/01/2018	−8	21	20/67	20/25	2
4	839M	1S1706750238	M/46	07/02/2018	−4	21	20/100	20/25	3

IOL = intraocular lens; BCVA = best corrected visual acuity; ^*∗*^time interval between the delivery and the operation.

## References

[1] Anis A. Y. (1982). Principles and evolution of intraocular lens implantation. *International Ophthalmology Clinics*.

[2] Abela-Formanek C., Amon M., Schild G., Schauersberger J., Heinze G., Kruger A. (2002). Uveal and capsular biocompatibility of hydrophilic acrylic, hydrophobic acrylic, and silicone intraocular lenses. *Journal of Cataract & Refractive Surgery*.

[3] Hollick E. J., Spalton D. J., Ursell P. G. (1999). Surface cytologic features on intraocular lenses. *Archives of Ophthalmology*.

[4] Özyol P., Özyol E., Karel F. (2017). Biocompatibility of intraocular lenses. *Türk Oftalmoloji Dergisi*.

[5] Tyagi P., Shah N., Jabir M. (2011). Intraoperative clouding of a posterior chamber intraocular lens. *International Ophthalmology*.

[6] Gauthier J. H., Pohl P. I. (2011). A general framework for modeling growth and division of mammalian cells. *BMC Systems Biology*.

[7] Kim D. J., Chuck R. S., Lee J. K., Park C. Y. (2017). Reversible opacification of hydrophobic acrylic intraocular lens-two cases report. *BMC Ophthalmology*.

[8] Mamalis N., Brubaker J., Davis D., Espandar L., Werner L. (2008). Complications of foldable intraocular lenses requiring explantation or secondary intervention-2007 survey update. *Journal of Cataract & Refractive Surgery*.

[9] Sher J. H., Gooi P., Dubinski W., Brownstein S., El-Defrawy S., Nash W. A. (2008). Comparison of the incidence of opacification of hydroview hydrogel intraocular lenses with the ophthalmic viscosurgical device used during surgery. *Journal of Cataract & Refractive Surgery*.

[10] Werner L. (2007). Causes of intraocular lens opacification or discoloration. *Journal of Cataract & Refractive Surgery*.

[11] Pandey S. K., Werner L., Apple D. J., Gravel J. P. (2002). Calcium precipitation on the optical surfaces of a foldable intraocular lens: a clinicopathological correlation. *Archives of Ophthalmology*.

[12] Miyake G., Ota I., Miyake K., Zako M., Iwaki M., Shibuya A. (2015). Late-onset toxic anterior segment syndrome. *Journal of Cataract & Refractive Surgery*.

[13] Suzuki T., Ohashi Y., Oshika T. (2015). Outbreak of late-onset toxic anterior segment syndrome after implantation of one-piece intraocular lenses. *American Journal of Ophthalmology*.

[14] Huang Y., Xie L. (2011). Delayed postoperative opacification of foldable hydrophilic acrylic intraocular lenses. *Journal of Biomedical Materials Research Part B: Applied Biomaterials*.

[15] Dai Y., Huang Y., Liu T., Xie L. (2014). Laboratory analyses of two explanted hydrophobic acrylic intraocular lenses. *Indian Journal of Ophthalmology*.

[16] Izak A. M., Werner L., Pandey S. K., Apple D. J. (2003). Calcification of modern foldable hydrogel intraocular lens designs. *Eye*.

[17] Neuhann I., Werner L., Izak A. (2004). Late postoperative opacification of a hydrophilic acrylic (hydrogel) intraocular lens: a clinicopathological analysis of 106 explants. *Ophthalmology*.

[18] Tehrani M., Mamalis N., Wallin T. (2004). Late postoperative opacification of memory lens hydrophilic acrylic intraocular lenses case series and review. *Journal of Cataract & Refractive Surgery*.

[19] Lee C. E., Kim Y. C., Chang S. D. (2010). Opacification of the optic of an akreos adapt intraocular lens. *Korean Journal of Ophthalmology*.

[20] Bompastor-Ramos P., Póvoa J., Lobo C. (2016). Late postoperative opacification of a hydrophilic-hydrophobic acrylic intraocular lens. *Journal of Cataract & Refractive Surgery*.

[21] Gurabardhi M., Haberle H., Aurich H., Werner L., Pham D.-T. (2018). Serial intraocular lens opacifications of different designs from the same manufacturer: clinical and light microscopic results of 71 explant cases. *Journal of Cataract and Refractive Surgery*.

[22] Park D. I., Ha S. W., Park S. B., Lew H. (2011). Hydrophilic acrylic intraocular lens optic opacification in a diabetic patient. *Japanese Journal of Ophthalmology*.

